# Genetic Analysis of Pathways Regulated by the von Hippel-Lindau Tumor Suppressor in Caenorhabditis elegans

**DOI:** 10.1371/journal.pbio.0020289

**Published:** 2004-09-07

**Authors:** Tammie Bishop, Kah Weng Lau, Andrew C. R Epstein, Stuart K Kim, Min Jiang, Delia O'Rourke, Christopher W Pugh, Jonathan M Gleadle, Martin S Taylor, Jonathan Hodgkin, Peter J Ratcliffe

**Affiliations:** **1**The Henry Wellcome Building of Genomic Medicine, University of OxfordOxfordUnited Kingdom; **2**Department of Developmental Biology and Genetics, Stanford University Medical CenterStanford, CaliforniaUnited States of America; **3**Department of Biochemistry, University of OxfordOxfordUnited Kingdom

## Abstract

The von Hippel-Lindau (VHL) tumor suppressor functions as a ubiquitin ligase that mediates proteolytic inactivation of hydroxylated α subunits of hypoxia-inducible factor (HIF). Although studies of VHL-defective renal carcinoma cells suggest the existence of other VHL tumor suppressor pathways, dysregulation of the HIF transcriptional cascade has extensive effects that make it difficult to distinguish whether, and to what extent, observed abnormalities in these cells represent effects on pathways that are distinct from HIF. Here, we report on a genetic analysis of HIF-dependent and -independent effects of VHL inactivation by studying gene expression patterns in *Caenorhabditis elegans.* We show tight conservation of the HIF-1/VHL-1/EGL-9 hydroxylase pathway. However, persisting differential gene expression in *hif-1* versus *hif-1; vhl-1* double mutant worms clearly distinguished HIF-1–independent effects of VHL-1 inactivation. Genomic clustering, predicted functional similarities, and a common pattern of dysregulation in both *vhl-1* worms and a set of mutants *(dpy-18, let-268, gon-1, mig-17,* and *unc-6),* with different defects in extracellular matrix formation, suggest that dysregulation of these genes reflects a discrete HIF-1–independent function of VHL-1 that is connected with extracellular matrix function.

## Abstract 2

Besides its known function of inactivating hypoxia-inducible factor (HIF), genetically engineered worms clearly demonstrate that there exist HIF-independent effects of the von Hippel- Lindau tumor suppressor

## Introduction

The von Hippel-Lindau *(VHL)* gene is a tumor suppressor that is mutated in the majority of both hereditary and sporadic, clear-cell renal carcinomas (Kaelin 2002). In hereditary VHL disease affected individuals are also predisposed to pheochromocytomas and retinal/central nervous system hemangioblastomas and develop multiple benign lesions in the kidney and other organs. Despite more than a decade of intensive investigation following identification of the defective gene in 1993 (Latif et al. 1993), the nature of the VHL tumor suppressor mechanism and how it relates to the physiological function of VHL remains unclear (Kaelin 2002).

To date, the best-understood function of VHL is as a ubiquitin ligase that affects oxygen-dependent proteolytic targeting of the α subunits of hypoxia-inducible factor (HIF) (Maxwell et al. 1999; Ohh et al. 2000). Oxygen-dependent hydroxylation of two HIF-α prolyl residues by HIF prolyl hydroxylases (Epstein et al. 2001; Ivan et al. 2001; Jaakkola et al. 2001) promotes interaction with VHL and targets HIF-α for degradation by the ubiquitin-proteasome pathway. In VHL-defective cells HIF-α subunits are stabilized and HIF is constitutively activated, resulting in the upregulation of HIF target genes (Maxwell et al. 1999). Whether this, or other putative VHL pathways, accounts for the tumor suppressor action is the subject of active investigation (Kondo et al. 2002, 2003; Maranchie et al. 2002). For instance, a number of different VHL-dependent cellular phenotypes have been defined by contrasting VHL-defective cells with transfectants re-expressing wild-type VHL (Kaelin 2002). These have highlighted effects of VHL on invasiveness, branching morphogenesis, and matrix assembly (Ohh et al. 1998; Koochekpour et al. 1999; Davidowitz et al. 2001; Kamada et al. 2001; Esteban-Barragan et al. 2002). However, mechanistic links to VHL function have not yet been defined and it is unclear whether or not these effects are secondary to dysregulation of HIF. This has led to attempts to define the existence, or otherwise, of non-HIF, VHL-regulated pathways by comparing patterns of gene expression induced by VHL inactivation with those induced by hypoxia (Wykoff et al. 2000; Zatyka et al. 2002; Y. Jiang et al. 2003). The observed patterns are not fully concordant, suggesting that there may be non-HIF, VHL-regulated pathways. However, these studies leave important uncertainties since HIF dysregulation might have secondary effects on pathways that are not themselves responsive to hypoxia and VHL might target hypoxia pathways other than HIF.

To address this we have used a genetic approach in *Caenorhabditis elegans.* Whereas mammalian cells possess three HIF-α isoforms that are targeted by VHL, C. elegans has a single HIF-α homolog (HIF-1) and a single VHL homolog (VHL-1), simplifying the genetic approach (Epstein et al. 2001; H. Jiang et al. 2001). Since homozygous *vhl-1* and *hif-1* loss-of-function worms are viable, we created *hif-1; vhl-1* worms and compared the effects of *vhl-1* inactivation on gene expression in wild-type and HIF-1–defective backgrounds. Our results clearly demonstrate the existence of both HIF-dependent and HIF-independent pathways of VHL-dependent gene expression. HIF-1–dependent effects of *vhl-1* inactivation on gene expression were also produced by inactivation of the HIF prolyl hydroxylase homolog EGL-9. In contrast, the HIF-1–independent effects of *vhl-1* inactivation were not observed in *egl-9* loss-of-function worms but were seen in a panel of mutant worms *(dpy-18, let-268, gon-1, mig-17,* and *unc-6)* bearing defects in genes involved in extracellular matrix function, supporting the existence of a conserved non-HIF pathway connecting VHL with an as yet unknown extracellular matrix function.

## Results

### Effect of VHL-1 Inactivation on Gene Expression in C. elegans

**Figure 1:**
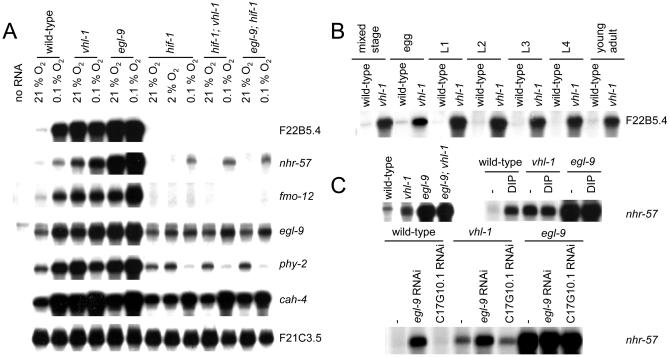
HIF-1–Dependent Effects of VHL-1 Inactivation Representative RNase protection assays of genes that were differentially expressed in the *vhl-1* versus wild-type microarray in (A) mixed-stage and (B) synchronized populations of worm. All genes are regulated by the VHL-1/HIF-1/EGL-9 pathway. (C) Regulation of *nhr-57* mRNA in *egl-9; vhl-1* worms and by *egl-9* RNAi and DIP in *vhl-1* worms. For RNAi experiments controls were L4440 vector alone (−) and C17G10.1, an irrelevant putative dioxygenase. F21C3.5 is a constitutively expressed gene used to control for RNA integrity. RNase protection assays were performed using worms cultured under normoxic conditions, unless otherwise indicated.

As a first step in defining VHL-1–dependent pathways in *C. elegans,* a whole-genome microarray was probed to compare transcript patterns in *vhl-1* versus wild-type worms (*n* = 1). From this array a set of genes (selected for amplitude of differential expression, signal intensity, quality of array signal, and putative function) was assayed quantitatively by ribonuclease (RNase) protection (Table 1). Of the 14 genes analyzed, six (F22B5.4, unknown function; *nhr-57,* predicted nuclear hormone receptor; *fmo-12,* predicted flavin monooxygenase; *egl-9,* HIF-1 prolyl hydroxylase [Epstein et al. 2001]; *phy-2,* procollagen prolyl 4-hydroxylase α subunit [Friedman et al. 2000]; and *cah-4,* predicted carbonic anhydrase) were strikingly downregulated by VHL-1 (Figure 1A; Table 2, column B). Further analysis in synchronized worm populations indicated that the VHL-1–dependent effects were observed in all stages (Figure 1B and unpublished data).

**Table 1:**
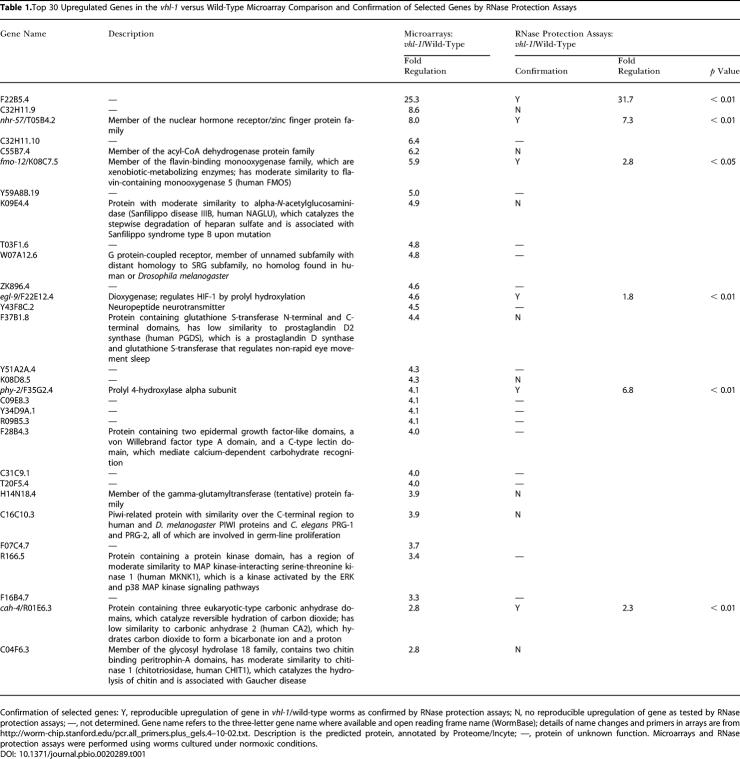
Top 30 Upregulated Genes in the *vhl-1* versus Wild-Type Microarray Comparison and Confirmation of Selected Genes by RNase Protection Assays

Confirmation of selected genes: Y, reproducible upregulation of gene in *vhl-1*/wild-type worms as confirmed by RNase protection assays; N, no reproducible upregulation of gene as tested by RNase protection assays; —, not determined. Gene name refers to the three-letter gene name where available and open reading frame name (WormBase); details of name changes and primers in arrays are from http://worm-chip.stanford.edu/pcr.all_primers.plus_gels.4–10-02.txt. Description is the predicted protein, annotated by Proteome/Incyte; —, protein of unknown function. Microarrays and RNase protection assays were performed using worms cultured under normoxic conditions

**Table 2:**
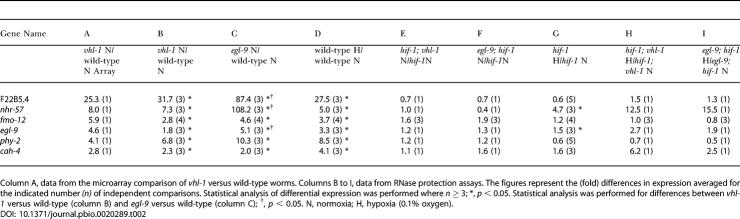
Differential Expression of VHL-1–Regulated Genes in Mutants Affecting the HIF-1/VHL-1/EGL-9 Pathway

Column A, data from the microarray comparison of *vhl-1* versus wild-type worms. Columns B to I, data from RNase protection assays. The figures represent the (fold) differences in expression averaged for the indicated number *(n)* of independent comparisons. Statistical analysis of differential expression was performed where *n* ≥ 3; *, *p* < 0.05. Statistical analysis was performed for differences between *vhl-1* versus wild-type (column B) and *egl-9* versus wild-type (column C); †, *p* < 0.05. N, normoxia; H, hypoxia (0.1% oxygen)

### Analysis of the EGL-9/HIF-1 Pathway

To determine the extent to which disruption of the conserved EGL-9/HIF-1 pathway mediates these effects we studied wild-type, *hif-1, vhl-1,* and *egl-9* single mutant worms and *hif-1; vhl-1* and *egl-9; hif-1* double mutant worms. Apart from the mild phenotype of the *vhl-1* worms (slightly uncoordinated, slow growth, and reduced brood size) and the egg-laying defective phenotype of *egl-9,* none of the worm strains showed obvious phenotypic abnormalities. Interestingly, *hif-1* corrected the phenotype of *egl-9.*

The findings indicate that all six genes are strongly regulated by the EGL-9/HIF-1 pathway (Figure 1A; Table 2). All six genes were inducible by hypoxia in wild-type worms (Table 2, column D) and strikingly upregulated in *egl-9* worms (Table 2, column C). *hif-1* inactivation abrogated the upregulation by the *egl-9* mutation (Table 2, columns C and F) and strikingly reduced induction by hypoxia (Table 2, columns D and G). Computational analysis revealed that five (F22B5.4, *nhr-57, fmo-12, egl-9,* and *cah-4*) of the six genes contained a potential HIF-1 binding core motif (RCGTG) within an arbitrarily defined region (−1,000 to +250 nucleotides) that was conserved in Caenorhabditis briggsae (Table 3), suggesting that these genes are direct HIF-1 transcriptional targets.

**Table 3:**
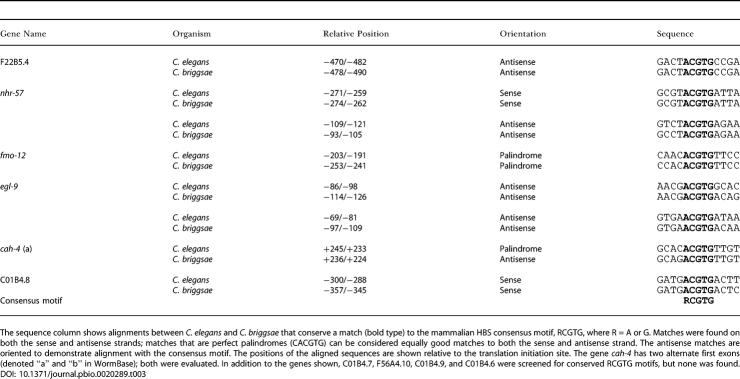
Evolutionarily Conserved HBS Consensus Sequences

The sequence column shows alignments between C. elegans and C. briggsae that conserve a match (bold type) to the mammalian HBS consensus motif, RCGTG, where R = A or G. Matches were found on both the sense and antisense strands; matches that are perfect palindromes (CACGTG) can be considered equally good matches to both the sense and antisense strand. The antisense matches are oriented to demonstrate alignment with the consensus motif. The positions of the aligned sequences are shown relative to the translation initiation site. The gene *cah-4* has two alternate first exons (denoted “a” and “b” in WormBase); both were evaluated. In addition to the genes shown, C01B4.7, F56A4.10, C01B4.9, and C01B4.6 were screened for conserved RCGTG motifs, but none was found

Though the six genes all conformed to the above patterns to demonstrate regulation by the EGL-9/HIF-1 pathway (Figure 1A; Table 2), there were differences. First, for some genes (F22B5.4, *nhr-57,* and *fmo-12*) expression in normoxia was entirely dependent on HIF-1, whereas other genes retained substantial normoxic expression in *hif-1* worms (Figure 1A). Second, three genes *(nhr-57, egl-9,* and *cah-4)* showed modest upregulation, and one gene *(phy-2)* showed modest downregulation, by hypoxia that was independent of HIF-1, VHL-1, and EGL-9 (Table 2, columns G–I). Finally, for certain genes, upregulation was clearly greater in *egl-9* than *vhl-1* worms, results being particularly striking for *nhr-57* (Table 2, columns C and B). To pursue this, we created *egl-9; vhl-1* double mutants and also exposed *vhl-1* worms to *egl-9* RNAi. Both procedures increased *nhr-57* expression, indicating that EGL-9 has non–VHL-1–mediated effects on this pathway (Figure 1C). Interestingly, the effects of genetic inactivation of *egl-9* in the *vhl-1* background were not mimicked by the dioxygenase inhibitor 2,2′-dipyridyl (DIP), suggesting that the VHL-1–independent repressive effects on *nhr-57* may be nonenzymatic.

### Evidence for a VHL-1–Dependent, HIF-1–Independent Pathway

**Figure 2:**
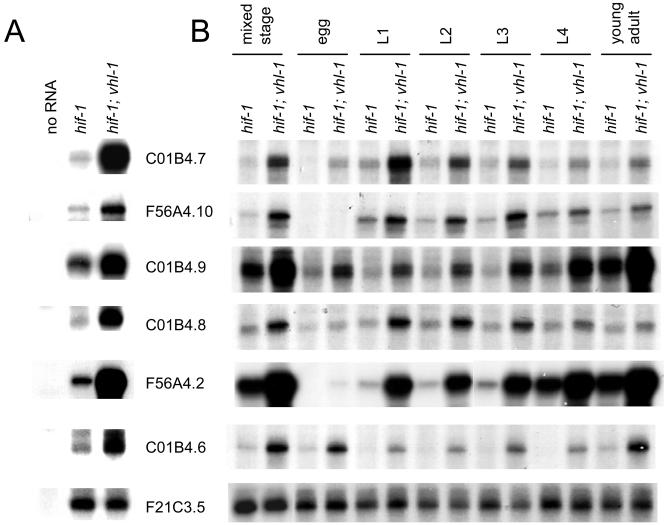
HIF-1–Independent Effects of VHL-1 Inactivation RNase protection assays of genes that were differentially expressed in the *hif-1; vhl-1* versus *hif-1* microarrays in (A) mixed-stage and (B) synchronized populations of worm. The results confirm the existence of VHL-1–dependent, HIF-1–independent effects on gene expression.

To address directly whether HIF-1–independent, VHL-1–mediated pathways exist, we performed further microarray comparisons of RNA from *hif-1; vhl-1* and *hif-1* worms (*n* = 3). Fewer genes showed differential expression than in the *vhl-1* versus wild-type array; however, persisting differential expression did suggest the existence of VHL-1 pathways that are independent of HIF-1 (Table 4). To test this, a number of genes were selected for further validation by RNase protection assay on the basis of amplitude of differential expression, *p* value, signal intensity, and quality of array signal. Of the 25 genes analyzed by RNase protection assay (Table 4), six (C01B4.7, F56A4.10, C01B4.9, and C01B4.8, all predicted transmembrane proteins belonging to the major facilitator superfamily [InterPro: IPR007114 and IPR005828]; F56A4.2, a predicted C-type lectin [InterPro: IPR001304]; and C01B4.6, a predicted aldose epimerase [InterPro: IPR008183]) showed clear downregulation by VHL-1 in a HIF-1–independent manner (Figure 2A; Table 5, column C). These effects were observed across essentially all developmental stages of the worm (Figure 2B). Computational analysis revealed that only one (C01B4.8) of the five HIF-1–independent, VHL-1–dependent genes tested (C01B4.7, F56A4.10, C01B4.9, C01B4.8, and C01B4.6; no single ortholog of F56A4.2 could be identified in C. briggsae) contained a potential HIF-1 binding site (HBS) within an arbitrarily defined region that was conserved in C. briggsae (see Table 3). This contrasts with the HIF-1–dependent, VHL-1–dependent genes validated by RNase protection assay (see Figure 1A), for which potential HBSs could be defined for five of the six genes tested (see Table 3).

**Table 4:**
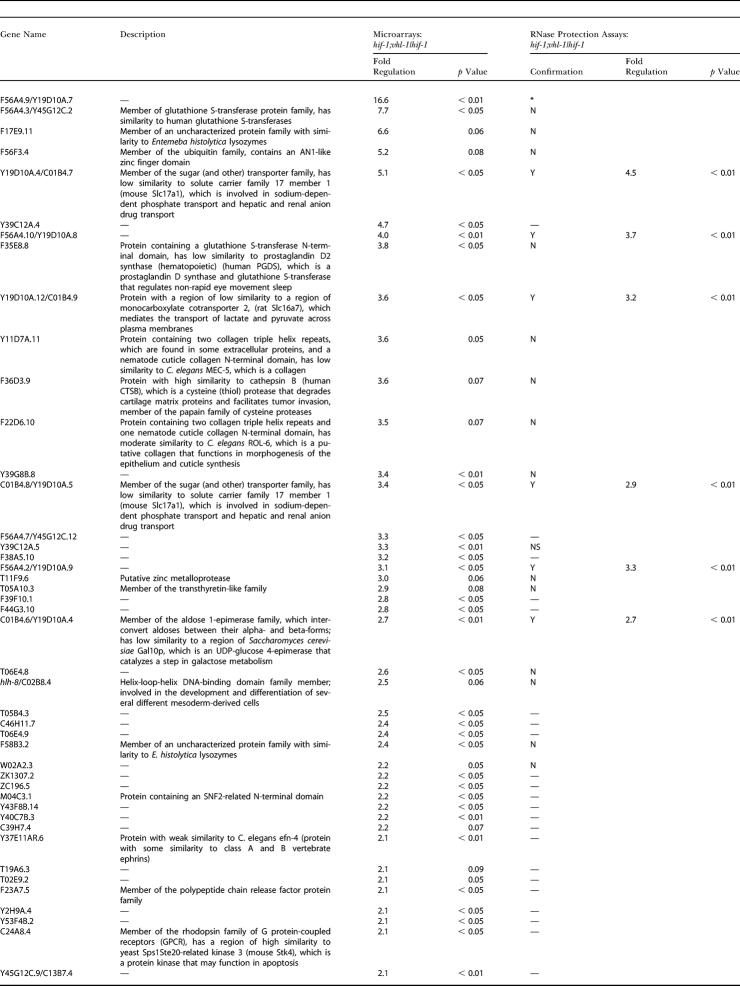
Upregulated Genes in the *hif-1; vhl-1* versus *hif-1* Microarray Comparisons and Confirmation of Selected Genes by RNase Protection Assays

Confirmation of selected genes: Y, reproducible upregulation of gene in *hif-1; vhl-1*/*hif-1* worms as confirmed by RNase protection assays; N, no reproducible upregulation of gene as tested by RNase protection assays; asterisk, not assayed, riboprobe could not be constructed; NS, no signal by RNase protection assay; —, not determined. Microarrays and RNase protection assays were performed using worms cultured under normoxic conditions. C35B8.1, C46A5.3, R03D7.5, T11F9.8, and ZK1010.7 were also tested by RNase protection assay based on microarray data; these genes did not show reproducible upregulation by RNase protection assays

**Table 5:**
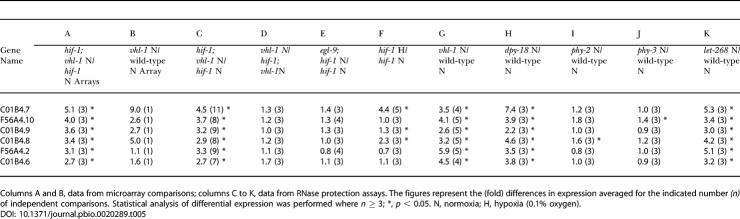
Differential Expression of HIF-1–Independent, VHL-1–Regulated Genes in Mutants Affecting the HIF-1/VHL-1/EGL-9 Pathway and Procollagen Hydroxylases

Columns A and B, data from microarray comparisons; columns C to K, data from RNase protection assays. The figures represent the (fold) differences in expression averaged for the indicated number *(n)* of independent comparisons. Statistical analysis of differential expression was performed where *n* ≥ 3; *, *p* < 0.05. N, normoxia; H, hypoxia (0.1% oxygen)

**Figure 3:**
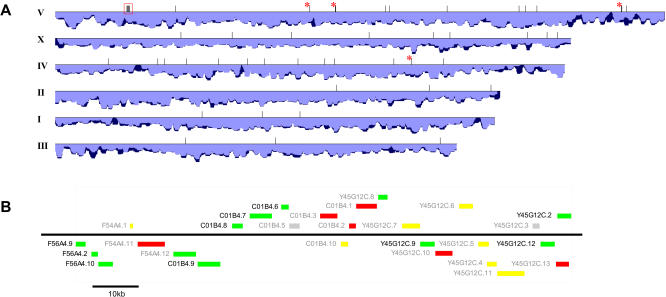
Chromosomal Clustering of VHL-1–Dependent (HIF-1–Independent) Genes (A) Chromosomal localization of VHL-1–dependent, HIF-1–independent genes. The positions of the genes from Table 4 are indicated by vertical ticks along the C. elegans chromosomes (shown to scale). Where two such genes are too close to be clearly resolved, the tick is marked by an asterisk. The single significant spatial clustering of VHL-1–dependent, HIF-1–independent genes is indicated by a red rectangle. The histogram under each chromosome shows the gene density (deeper bar, greater density) calculated as a sliding window of 100,000 bp moving with 10,000-bp increments along each chromosome. Dark blue indicates total annotated gene density, and light blue indicates the density of genes from the microarray that passed preliminary quality control. (B) Organization of the VHL-1–regulated (HIF-1–independent) gene cluster from Chromosome V. The relative positions and sizes of gene transcription units are shown to scale, with genes transcribed left to right above the horizontal line and right to left below the line. Names in black indicate genes that passed all selection criteria to be considered upregulated in *hif-1; vhl-1* versus *hif-1* worms (see Table 4). Genes with a mean >2.0-fold upregulation are indicated by green boxes, 1.5- to 2-fold are yellow, and <1.5-fold are red. Genes for which no data were obtained are shown as light grey.

Interestingly, all six genes validated by RNase protection assay to be negatively regulated by VHL-1 in a HIF-1–independent manner localize within 45 kb on Chromosome V (although they were not situated in physical proximity on the array). We applied single-linkage clustering (nearest-neighbor method) (Sneath 1957; Dillon and Goldstein 1984; Roy et al. 2002) to identify spatial clusters of genes considered to be negatively regulated by VHL-1 in a HIF-1–independent manner from the microarray data (Table 4) and random sampling to evaluate the significance of such clusters. Using a clustering threshold of 96,985 bp (see Materials and Methods), one cluster of ten genes and four clusters of two genes were identified (Figure 3A). On 100,000 simulated datasets of 57 randomly selected genes (equal number to that of VHL-1–dependent, HIF-1–independent genes; Table 4), the frequency of observed cluster sizes was as follows: one gene, 5,043,442; two genes, 298,425; three genes, 18,198; four genes, 1,190; five genes, 66; six genes, 4. No clusters of more than six genes were observed. Therefore, the cluster of ten VHL-1–regulated (HIF-1–independent) genes, which extends over 110 kb to include F56A4.9, Y45G12C.9, Y45G12C.12, and Y45G12C.2 in addition to the six genes validated by RNase protection assays, can be considered statistically significant to *p* ≪ 10^−5^ (Figure 3B). Recent C. elegans genomic assemblies (for example, WS120) have shown that the entire 110-kb region containing the coregulated gene cluster is arranged in tandem with a second nearly identical segmental duplication of the locus (>99.9% identical in alignment). At this level of identity, our microarray and RNase protection analyses cannot discriminate between the two copies of each gene, so for all of our analyses we have only used the names of the distal copy and genes from the proximal copy were excluded from computational analyses.

**Table 6:**
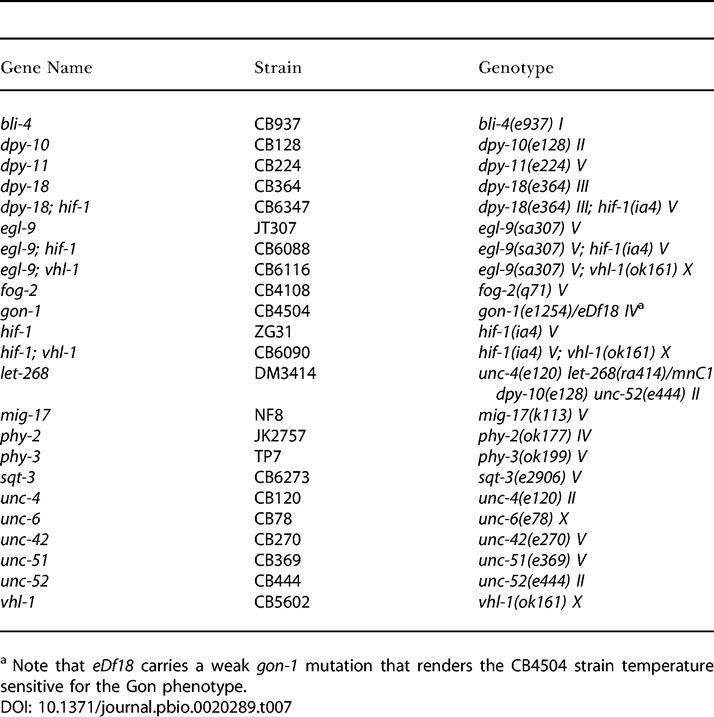
C. elegans Strains and Alleles

^a^ Note that *eDf18* carries a weak *gon-1* mutation that renders the CB4504 strain temperature sensitive for the Gon phenotype

### Extracellular Matrix Link to Novel VHL-1 Pathway

**Figure 4:**
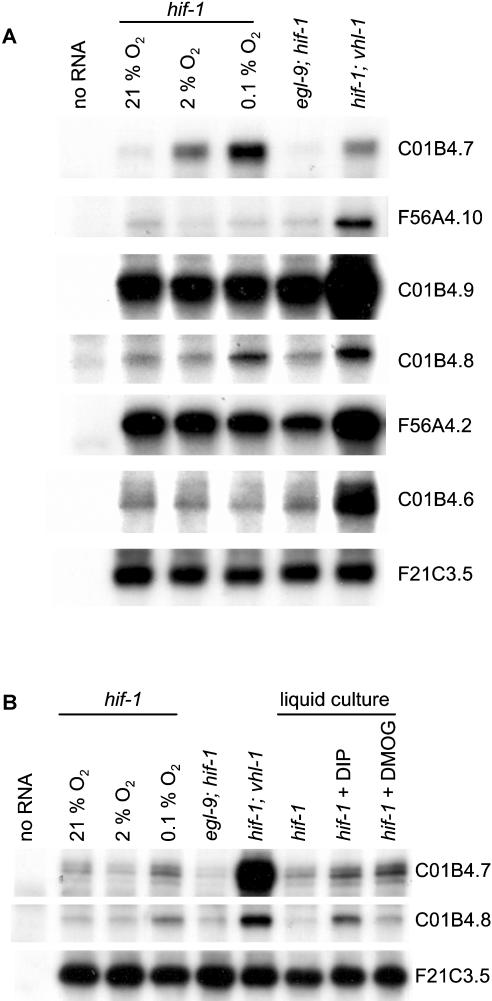
Responses of VHL-1–Dependent, HIF-1–Independent Genes to *egl-9* Inactivation, Hypoxia, and 2-Oxoglutarate Dioxygenase Inhibitors RNase protection assays showing regulation of VHL-1–dependent, HIF-1–independent genes by (A) EGL-9 and hypoxia and (B) pharmacological inhibitors of 2-oxoglutarate dioxygenases: DIP and DMOG. None of the genes is regulated by EGL-9, but two genes (C01B4.7 and C01B4.8) show modest induction by hypoxia, DIP, and DMOG.

**Figure 5:**
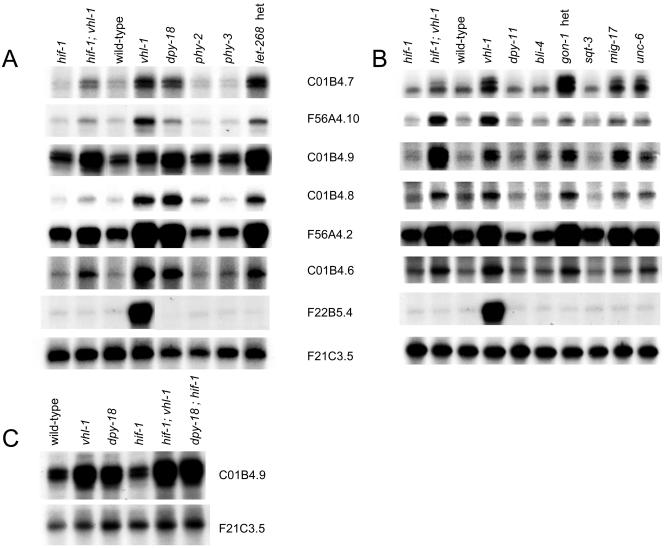
Sensitivity of VHL-1–Regulated Genes to Defects in Extracellular Matrix-Associated Proteins RNase protection assays showing altered expression of VHL-1–regulated genes that are HIF-1 independent (upper six panels) and HIF-1 dependent (F22B5.4) in worms bearing mutations affecting (A) procollagen prolyl and lysyl hydroxylases and (B) other extracellular matrix-associated proteins. A common pattern of upregulation is observed in *hif-1; vhl-1, vhl-1, dpy-18, let-268, gon-1, mig-17,* and *unc-6* worms but not other mutants. This contrasts with the HIF-1–dependent gene F22B5.4, which is upregulated in *vhl-1* worms but none of the other mutants. (C) RNase protection assay for C01B4.9 illustrating DPY-18–mediated changes in expression that are independent of HIF-1.

Since ubiquitin ligases commonly recognize more than one substrate, we considered whether these HIF-1–independent genes might be regulated by prolyl hydroxylation of another VHL-1 substrate by EGL-9. However, this was not supported by any differential expression in *egl-9; hif-1* versus *hif-1* worms (Figure 4A; Table 5, column E). Nevertheless, two genes, C01B4.7 and C01B4.8, were upregulated in *hif-1* worms by hypoxia and the 2-oxoglutarate dioxygenase inhibitors, DIP and dimethyloxalylglycine (DMOG) (Figure 4; Table 5, column F), suggesting that another enzyme in this class might be involved. The procollagen prolyl hydroxylases DPY-18, PHY-2, and PHY-3 (Friedman et al. 2000; Riihimaa et al. 2002) and the procollagen lysyl hydroxylase LET-268 (Norman and Moerman 2000) were tested as potential candidates. A clear pattern was observed. All six VHL-1–regulated, HIF-1–independent genes were reproducibly downregulated by DPY-18 and LET-268 but not by PHY-2 or PHY-3 (Figure 5A; Table 5, columns H–K). The strain carrying the heterozygous *let-268* mutation is heterozygous for *unc-4, dpy-10,* and *unc-52;* however, the VHL-1–dependent, HIF-1–independent genes were not differentially expressed in *unc-4, dpy-10,* or *unc-52* worms, indicating that the effects were due to LET-268 (unpublished data). Further experiments on *dpy-18; hif-1* double mutant worms clearly indicated that the effects of DPY-18 on this group of genes were (like the effects of VHL-1) HIF-1 independent (Figure 5C and unpublished data).

Downregulation by DPY-18 and LET-268 is consistent with the positive effects of hypoxia, DIP, and DMOG, since all these stimuli inhibit DPY-18 and LET-268. However, the involvement of a lysyl, as well as a prolyl, hydroxylase suggests that the effects were unlikely to arise from failure of hydroxylation of a second prolyl hydroxylation substrate recognized by VHL-1 and were more likely to be related to a common function of DPY-18 and LET-268, such as a function in extracellular matrix formation. To pursue this, we tested the effects of defects in proteins involved in other aspects of extracellular matrix formation (either in the cuticle or basement membrane) that are distinct from protein hydroxylation. These experiments indicated that the six genes were, to varying extents, upregulated in the basement membrane-associated *gon-1* (heterozygote), *mig-17,* and *unc-6* mutant worms but not in the cuticle-associated *dpy-11, bli-4,* or *sqt-3* mutant worms (Figure 5B and unpublished data). In contrast, none of the HIF-1–dependent genes was upregulated in these mutants (Figure 5B and unpublished data). GON-1 and MIG-17 encode secreted metalloproteases and UNC-6 encodes a netrin; all are thought to be involved in basement membrane remodeling/cell migration during gonadal morphogenesis (Hedgecock et al. 1990; Blelloch and Kimble 1999; Nishiwaki et al. 2000). Conversely, DPY-11 (a thioredoxin) and BLI-4 (a serine endoprotease) are both involved in collagen formation in the worm cuticle (Thein et al. 2003) and SQT-3 encodes a cuticular collagen. These results therefore extend the characterization of the VHL-1–dependent, HIF-1–independent pathway and support a connection with extracellular matrix/basement membrane function.

## Discussion

By comparing the effects of *vhl-1* inactivation in different genetic backgrounds, these data clearly distinguish HIF-1–dependent and –independent effects of VHL-1 on gene expression. Somewhat surprisingly, all of the VHL-regulated genes analyzed fell into one of two patterns: independent of HIF-1 and EGL-9 and dependent on DPY-18, LET-268, GON-1, MIG-17, and UNC-6, or the reverse, suggesting that they reflect perturbation of two discrete aspects of VHL-1 function.

The HIF-1–dependent expression pattern of all six genes chosen for detailed analysis from the *vhl-1* versus wild-type array underlines the importance of the HIF-1 pathway in VHL-1 function. Computational analysis revealed that five of these genes (F22B5.4, *nhr-57, fmo-12, egl-9,* and *cah-4*) have at least one HIF-1 binding core motif (RCGTG) that is conserved in C. briggsae within an arbitrarily defined (−1,000 to +250 nucleotides) promoter region, suggesting that they are direct HIF-1 transcriptional targets. Several genes (*egl-9,* HIF prolyl hydroxylase; *phy-2,* procollagen prolyl 4-hydroxylase α subunit; and *cah-4,* carbonic anhydrase) have mammalian homologs that are HIF targets (Ivanov et al. 1998; Takahashi et al. 2000; Epstein et al. 2001), emphasizing the extent of conservation of the pathway. Others, such as flavin monooxygenase *fmo-12* and the nuclear hormone receptor *nhr-57,* are apparently novel HIF-1 target genes. Interestingly, some of these HIF-1–dependent genes were partly downregulated by EGL-9 in a VHL-1– and iron-independent manner, suggesting that, in addition to the HIF-1/VHL-1 pathway, EGL-9 regulates HIF-1 transcriptional activity via a novel pathway.

Remarkably, among the candidate genes tested from the *hif-1; vhl-1* versus *hif-1* screens, all six that showed reproducible (HIF-1–independent) regulation by VHL-1 were located within 45 kb on Chromosome V. Analysis of the microarray data revealed that there was indeed a single, highly significant (*p* < 10^−5^) chromosomal cluster of genes negatively regulated by VHL-1 in a HIF-1–independent manner and that in total this cluster extended over 110 kb to include F56A4.9, Y45G12C.9, Y45G12C.12, and Y45G12C.2 in addition to the six genes validated by RNase protection assay. The chromosomal localization of genes in C. elegans is not random, with functionally related genes located close to one another (Roy et al. 2002) or even organized into operons (Blumenthal and Gleason 2003). Even though, based on the absence of spliced leader SL2 sequences (Blumenthal et al. 2002) and the presence of inverse transcriptional orientations, the genes in this cluster do not appear to be within the same operon, there may be a functional relevance to their physical proximity. Four of the six genes validated by RNase protection assay (C01B4.7, F56A4.10, C01B4.9, and C01B4.8) encode membrane transporters of the major facilitator superfamily, a family of transporters involved in passive transport of small solutes. C01B4.9 clusters phylogenetically with monocarboxylate transporters, and C01B4.7, F56A4.10, and C01B4.8 cluster with sodium phosphate transporters (unpublished data). Both gene families have been subject to rounds of gene duplication in the vertebrate and nematode lineages. As such, it is not possible to define one-to-one orthologous relationships for these genes between C. elegans and *Homo sapiens.* Nevertheless, the genomic clustering, predicted functional similarities, and common pattern of perturbed expression across an extensive set of mutant worms suggest that the upregulation of the genes in *vhl-1* worms reflects the disturbance of a specific function of VHL-1.

The common effects of inactivating mutations in *vhl-1* and in genes that manifest functional overlap in extracellular matrix formation—*dpy-18* and *let-268* (encoding procollagen hydroxylases) (Friedman et al. 2000; Norman and Moerman 2000), *gon-1* and *mig-17* (encoding secreted metalloproteases) (Blelloch and Kimble 1999; Nishiwaki et al. 2000), and *unc-6* (encoding the extracellular guidance protein, netrin) (Hedgecock et al. 1990)—suggest a related function for this HIF-1–independent VHL pathway. Interestingly, VHL-defective renal carcinoma cells demonstrate a variety of matrix-related abnormalities, including abnormal fibronectin assembly, defective formation of fibrillar adhesions, and changes in branching morphogenesis and migration (Ohh et al. 1998; Koochekpour et al. 1999; Davidowitz et al. 2001; Kamada et al. 2001; Esteban-Barragan et al. 2002). These abnormalities can be corrected by transfection of renal carcinoma cells with wild-type *vhl,* indicating that they are attributable, either directly or indirectly, to VHL loss of function. Furthermore, immunoprecipitation studies using renal carcinoma cell extracts have indicated that VHL binds to fibronectin (Ohh et al. 1998). Most tumor-associated VHL mutants, when transfected into VHL-defective renal carcinoma cells, are defective in both complementing HIF dysregulation and fibronectin binding (Kaelin 2002). However, mutations associated with type 2C (predisposition to pheochromocytoma only) VHL disease complement defective HIF regulation but bind fibronectin with lower affinity than wild-type VHL (Hoffman et al. 2001). Though the precise link to abnormal matrix assembly remains unclear, this has suggested a HIF-independent function of VHL. The present study supports the existence of a HIF-independent pathway connected with extracellular matrix function and suggests that this may be a highly conserved function of VHL that is potentially amenable to genetic analysis in model organisms.

## Materials and Methods

### 

#### Strains and culturing conditions.

Worms were studied as mixed-stage populations or as synchronized populations following brief exposure to sodium hypochlorite. Exposure to hypoxia (2% or 0.1% oxygen), DIP (200 μM), and DMOG (1 mM) was for 18 h (Epstein et al. 2001). RNA interference (RNAi) was performed by feeding worms Escherichia coli strain HT115(DE3) expressing double-stranded (ds) RNA on Nematode Growth Medium containing 1 mM isopropyl-β-D-thiogalactopyranoside (ITPG) and 50 μg/ml ampicillin for 72 h. Plasmids for ds RNA production were derivatives of the L4440 vector and were obtained from J. Ahringer (Cambridge, United Kingdom); ds RNA sequences are available on WormBase (http://www.wormbase.org). Wild-type worms were Bristol strain (N2); mutant strains were obtained from the *Caenorhabditis* Genetics Center (Table 6). Strains were maintained at room temperature except for the temperature-sensitive *gon-1* worms (maintained at 18 °C). The double mutants *hif-1; vhl-1, egl-9; hif-1,* and *dpy-18; hif-1* were constructed using either *fog-2* or *unc-51* to mark *hif-1*(+); the double mutant *egl-9; vhl-1* was constructed using *unc-42* to mark *egl-9*(+); PCR was used to confirm homozygosity.

#### Microarray screening.

Microarray comparisons of wild-type versus *vhl-1* worms and *hif-1* versus *hif-1; vhl-1* worms were performed on independent samples of RNA (*n* = 1 and 3, respectively), using near full-genome C. elegans DNA microarrays (M. Jiang et al. 2001). Total RNA was extracted from mixed-stage populations of worm cultured under normoxic conditions using Tri-reagent (Sigma, Poole, Dorset, United Kingdom) and mRNA purified using oligo-dT beads (Qiagen, Crawley, West Sussex, United Kingdom). cDNA synthesis and microarray hybridization and scanning were performed as described previously (M. Jiang et al. 2001). Cy5-dUTP was used to label cDNA from wild-type and *hif-1* worms and Cy3-dUTP was used to label cDNA from *vhl-1* and *hif-1; vhl-1* worms. The arrays were computer normalized by the default procedure in the Stanford Microarray Database (SMD); primary array data are available on the SMD (http://genome-www.stanford.edu/microarray) and are also shown in Tables S1 through S4. Fold change was calculated as the ratio of the means of Cy3-dUTP intensity to normalized Cy5-dUTP intensity (normalized to correct for signal differences between Cy3-dUTP and Cy5-dUTP intensities across the whole array) with median background intensities subtracted from both signal intensities to correct for the background (see SMD). Genes with background-corrected signal intensities below zero or with array spots that were flagged in the SMD as being unreliable were discarded as a preliminary quality control. For the *hif-1* versus *hif-1; vhl-1* microarray comparisons (*n* = 3) the log_2_ fold change was calculated as the mean of the three log_2_ transformed fold changes. To test for significant upregulation, the mean log_2_ fold change was compared with zero using a Student's *t* test. The genes were ranked by amplitude of fold upregulation and a subset of genes was selected for potential validation by RNase protection assays (see Tables 1 and 4) based on the following criteria: (a) *t* test, *p* < 0.10 (for the *hif-1* versus *hif-1; vhl-1* microarray comparisons, *n* = 3); (b) mean Cy3-dUTP and Cy5-dUTP background-corrected signal intensities exceeding 300 and 100 U, respectively (lower intensities than these were difficult to detect by RNase protection assay); and (c) high spot quality as judged by manual inspection. For the *hif-1* versus *hif-1; vhl-1* microarray comparisons (*n* = 3), genes (which had been filtered as described above) were considered to be differentially expressed if the mean fold change was greater than 2.0 (see Table 4).

#### RNase protection assays.

Assays were performed on total RNA from mixed-stage populations of worm cultured under normoxic conditions, unless otherwise indicated. Details of riboprobe templates are provided in Table 7; details of genes tested are shown in Tables 1 and 4. Quantification was performed using a phosphorimager (Molecular Dynamics, Sunnyvale, California, United States) and related to an internal control assay for the constitutively expressed F21C3.5 (protein with similarity to mouse prefoldin subunit 6). Where *n* ≥ 3, the log_2_ fold change was calculated from the mean of the log_2_ transformed fold changes and statistical significance was calculated by comparing the mean log_2_ fold change with zero using a Student's *t* test.

**Table 7:**
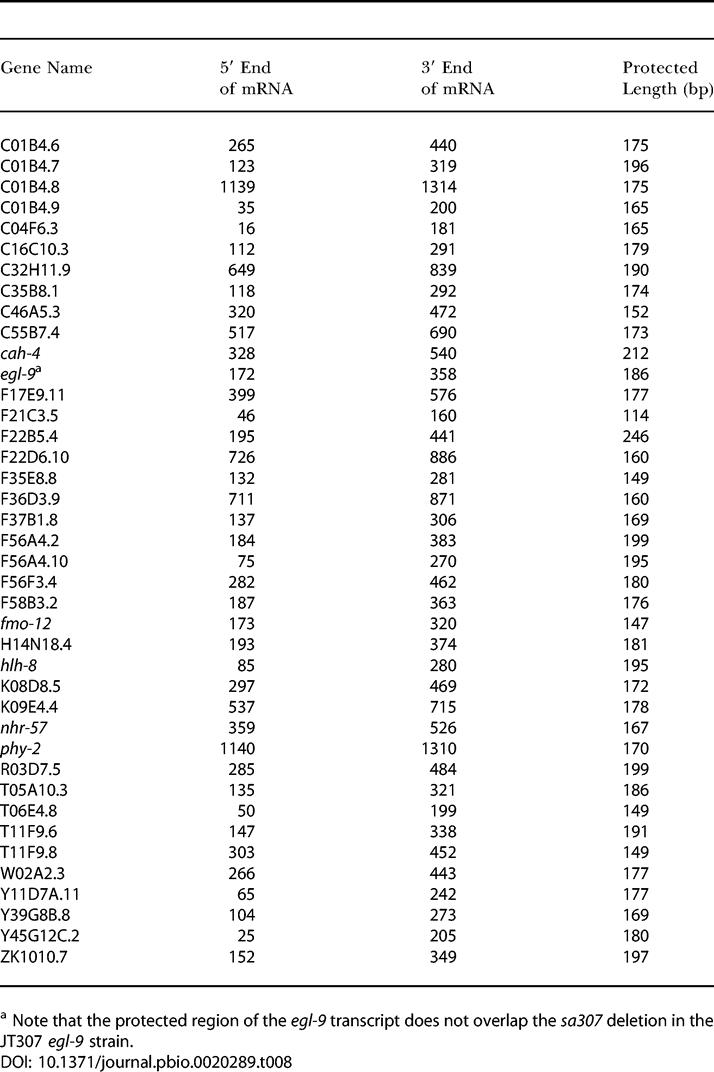
Sequence and Length of Riboprobes

^a^ Note that the protected region of the *egl-9* transcript does not overlap the *sa307* deletion in the JT307 *egl-9* strain

#### Computational analyses

(1) Identification of potential HBSs (see Table 3). Orthologs of C. elegans genes were identified in the C. briggsae genome assembly (cb25) as reciprocal best matches by BLASTN, initiated with the C. elegans gene coding sequence (a single ortholog of F56A4.2 could not be defined). Translation initiation sites (well-annotated surrogates for transcriptional start sites; none of these genes are annotated as having spliced 5′ UTRs) were inferred in both C. briggsae and C. elegans through alignment with the C. elegans coding sequence (WormBase, WS117). Sequences encompassing the 1,000 nucleotides upstream to 250 nucleotides downstream of the translation start sites for orthologous genes were aligned using DNA Block Aligner (Jareborg et al. 1999) with the following options: gap = 0.001 and blockopen = 0.005. Sequence alignments were searched with the HBS motif RCGTG (Camenisch et al. 2001), identifying cases where HBS-like motifs were conserved between both C. elegans and *C. briggsae.*

(2) Single-linkage analysis to determine spatial clusters of VHL-1–dependent, HIF-1–independent genes (see Figure 3). A maximum distance for the linking of two clusters was determined by ranking the distance between 10,000 randomly selected pairs of genes from the same chromosome (but sampled over all six nuclear chromosomes) and selecting as a threshold the first percentile of the distribution, 96,985 bp. Intergene distances were calculated from the closest point between the annotated coding sequence of each gene; genes on separate chromosomes were considered to have an infinite intergene distance. Simulations were performed using genes selected at random from genes that were represented on the microarray and that passed preliminary quality control criteria. All genomic coordinates were based on genomic assembly WS120 and the associated WormBase annotation of genes obtained from the University of California, Santa Cruz Genome Browser (http://genome.ucsc.edu/). The software used for simulations and clustering was implemented in Perl and is available on request.

## Supporting Information

Primary microarray data can be viewed at http://genome-www.stanford.edu/microarray.

Table S1 *vhl-1* versus Wild-Type Microarray Comparison Primary microarray data for the *vhl-1* (green, channel 1) versus wild-type (red, channel 2) comparison.
(6.5 MB XLS).

Table S2 *hif-1; vhl-1* versus *hif-1* Microarray Comparisons I Primary microarray data for the three independent *hif-1; vhl-1* (green, channel 1) versus *hif-1* (red, channel 2) microarray comparisons. Continued in Tables S3 and S4.
(6.6 MB XLS).

Table S3 *hif-1; vhl-1* versus *hif-1* Microarray Comparisons II Continuation of Table S2.
(6.6 MB XLS).

Table S4 *hif-1; vhl-1* versus *hif-1* Microarray Comparisons III Continuation of Tables S2 and S3.
(6.6 MB XLS).

### Accession Numbers

Primary array data have been deposited in ArrayExpress (http://www.ebi.ac.uk/arrayexpress/) under accession number E-SMDB-23.

The *H. sapiens VHL* gene discussed in this paper can be found in Online Mendelian Inheritance in Man (OMIM) under accession number 608537 (http://www.ncbi.nlm.nih.gov:80/entrez/dispomim.cgi?id=608537).

The C. elegans genes discussed in this paper (*bli-4,* C01B4.6, C01B4.7, C01B4.8, C01B4.9, *cah-4, dpy-10, dpy-11, dpy-18, egl-9,* F21C3.5, F22B5.4, F56A4.2, F56A4.9, F56A4.10, *fmo-12, fog-2, gon-1, hif-1, let-268, mig-17, nhr-57, phy-2, phy-3, sqt-3, unc-4, unc-6, unc-42, unc-51, unc-52, vhl-1,* Y45G12C.2, Y45G12C.9, and Y45G12C.12) can be found in the WormBase database by including the name of the gene at the end of the URL (e.g., for *bli-4,* http://wormbase.org/db/gene/gene?name=bli-4).

**Table 4:**
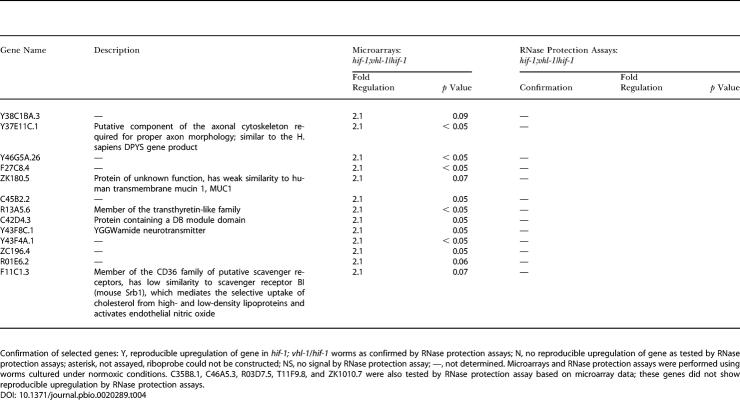
Continued

## Abbreviations

DIP: 2,2′-dipyridyl
DMOG: dimethyloxalylglycine
HBS: HIF-1 binding site
HIF: hypoxia-inducible factor
RNase: ribonuclease
SMD: Stanford Microarray Database
VHL: von Hippel-Lindau
